# Using natural language processing to explore heterogeneity in moral terminology in palliative care consultations 

**DOI:** 10.1186/s12904-021-00716-3

**Published:** 2021-01-25

**Authors:** Eline van den Broek-Altenburg, Robert Gramling, Kelly Gothard, Maarten Kroesen, Caspar Chorus

**Affiliations:** 1 University of Vermont, Robert Larner, M.D. College of Medicine, 89 Beaumont Avenue, Burlington, VT 05405 USA; 2 Delft University of Technology, Stevinweg 1, Delft, CB 2628 The Netherlands

**Keywords:** Palliative care, End-of-life, Conversation science, Morality, Decision-making, Natural language processing, Latent class analysis, Poisson regression

## Abstract

**Background:**

High quality serious illness communication requires good understanding of patients’ values and beliefs for their treatment at end of life. Natural Language Processing (NLP) offers a reliable and scalable method for measuring and analyzing value- and belief-related features of conversations in the natural clinical setting. We use a validated NLP corpus and a series of statistical analyses to capture and explain conversation features that characterize the complex domain of moral values and beliefs. The objective of this study was to examine the frequency, distribution and clustering of morality lexicon expressed by patients during palliative care consultation using the Moral Foundations NLP Dictionary.

**Methods:**

We used text data from 231 audio-recorded and transcribed inpatient PC consultations and data from baseline and follow-up patient questionnaires at two large academic medical centers in the United States. With these data, we identified different moral expressions in patients using text mining techniques. We used latent class analysis to explore if there were qualitatively different underlying patterns in the PC patient population. We used Poisson regressions to analyze if individual patient characteristics, EOL preferences, religion and spiritual beliefs were associated with use of moral terminology.

**Results:**

We found two latent classes: a class in which patients did not use many expressions of morality in their PC consultations and one in which patients did. Age, race (white), education, spiritual needs, and whether a patient was affiliated with Christianity or another religion were all associated with membership of the first class. Gender, financial security and preference for longevity-focused over comfort focused treatment near EOL did not affect class membership.

**Conclusions:**

This study is among the first to use text data from a real-world situation to extract information regarding individual foundations of morality. It is the first to test empirically if individual moral expressions are associated with individual characteristics, attitudes and emotions.

## Background

End-of-life (EOL) care causes many challenges for patients and palliative care (PC) physicians. Decisions that must be made regarding the care of the dying patient must be considered within the context of the psychological, physical, financial and social experiences of the patient’s life [1]. Within the hybrid of these domains, researchers have tried to identify which factors are most important to patients, how they relate to decisions and how decisions relate to PC treatments and outcomes [2–5]. Previous studies have analyzed the relationship between patient’s preferences and PC treatments [6]; between PC treatments and outcomes [2]; and between preferences and outcomes [3, 7, 8]. Treatments that are inconsistent with patient preferences are associated with some negative outcomes, such as higher healthcare utilization costs [6], lower quality of life, and physical and psychological distress [7]. Some PC research has also focused on the relation between underlying factors such as beliefs, norms and values and preferences, sometimes leading to EOL decisions [7–9]. Studies found that religion is one factor in a patient’s desire to request life-sustaining treatments even when a palliative care (PC) physician thinks such treatments are ineffective [10, 11]. Indeed, some religious laws prohibit interventions that shorten life, or reversely: interventions that extend life. Spiritual beliefs are also often documented in surveys and previous research has already explored the importance of these beliefs in seriously ill, hospitalized populations [8, 12–14].

In order to get a better understanding of some of these underlying factors, PC communication plays an important role in improving quality. The primary purpose of a PC consultation is to give patients information about their prognosis, as well as discuss goals of care, and address patients’ questions or concerns. Previous research has established that feeling “Heard & Understood” is a promising quality measure for PC communication in the inpatient palliative care setting [1–3], and that mirroring and reflection in the communication can help patients understand their prognosis better [4–6].

It is established that patients who have a better understanding of their prognosis are more likely to prefer and have treatments that are aligned with their personal values, goals and beliefs [15]. Palliative care consultations can thus be an important factor influencing palliative care outcomes, however, it remains unclear what defines a “quality” conversation or “effective” palliative care consultation. Based on previous studies, we know that informing patients about their prognosis is important [16–18], but the way physicians communicate these messages may also depend on how patients receive the information and willingness to listen. The latter, for example, has been shown to relate to dogmatism, among others [19]. The content and addressing uncertainty [9] is important, but using conversational pauses [20] and connectional silence [21] have also been proven to be effective tools in PC communication. Quality of PC consultations is primarily focused on aligning prognosis communication with patient’s personal values and beliefs to optimize understanding.

In this context, the choice of language plays an important role in PC consultations. It is well-established in sociolinguistics that the use of particular words in conversation are a reflection of underlying value [22–24]. Little is known, in the context of end-of-life care, about which factors may explain some of the rhetoric used by patients. In order for physicians to improve prognosis communication, we need to be able to differentiate PC consultations and get a better understanding of differences in rhetoric used. This way physicians may be able to better align conversation language with patient’s underlying values, beliefs and preferences in prognosis communication.

The objective of this study is to identify specific moral rhetoric used by patients in palliative care consultations and analyze if emotions, self-reported EOL preferences, religion and spiritual needs are associated with differences in moral expressions. We focus on analyzing moral rhetoric in PC consultations and explore the factors related to differences in moral expressions used by patients. The main research question of this study is: “Are preferences, emotions, religious affiliation and spiritual needs associated with vice or virtue and different moral expressions in EOL conversations?” This is a unique contribution to the literature as this study combines attitudinal data and moral expressions and maps how these are being used in a real-life and morally salient context.

### Foundations of morality

There has been some research into the role of morality in palliative care. Some scholars have described morality as one of the three sources of transcendence in PC patients^14^ and others have looked at whether these values or beliefs have an effect on PC outcomes, such as coping strategies, emotional outcomes and spiritual quality of life [25]. This potential influence of moral values makes the study of EOL decisions a unique and important contribution to empirical investigation of human motivations and decision making, capturing the full social-functional range of morality.

There are several schools of thought in moral psychology defining “morality”. The moral foundation theory (MFT), developed by Haidt and Joseph (2004), has been one of the most influential theories within moral psychology in the last decade. The MFT intends to explain the origins of and variation in human moral reasoning based on innate, modular foundations. In one of the key publications, Graham, Haidt and colleagues explain that “monists” describe morality as “one” type: this is usually identified as justice or fairness, referred to as “virtue” [26]. As time has evolved, evolutionary thinking has encouraged pluralist thinking about morality, they suggest. They describe in detail [11] how five moral foundations can be defined, which can be described by their characteristic emotions and relevant virtues:

1. The Care/harm foundation: compassion for victim; anger at perpetrator. Relevant virtues include caring and kindness.
2. The Fairness/cheating foundation: anger, gratitude, guilt. Relevant virtues include fairness, justice, trustworthiness.
3. The Loyalty/betrayal foundation: group pride, rage at traitors. Virtues: loyalty, patriotism, self-sacrifice.
4. The Authority/subversion foundation: based on respect and fear. Virtues include obedience and deference.
5. The Sanctity/degradation foundation: based on disgust, virtues include temperance, chastity, piety, cleanliness.

As can be seen, Graham and Haidt describe the five foundations that can have either a “vice” or a “virtue” realm. For example, loyalty is the “virtue” in the third foundations where betrayal is the “vice”. The authors describe the two different values, positive and negative, as being part of one and the same foundation that can be explained by group pride and rage at traitors.

Despite the ongoing work on this MFT in moral psychology, the validity of this scale (both internal and external) across different cultures is not yet fully established. Also, it remains a challenge for the theorists to fully capture the highly variable and subjective nature of individual moral values [27]. For this reason, Graham and Haidt developed a MFT dictionary which can be used to analyze any corpus of text. They recently “called” on researchers in big data analytics to use their dictionary [28] to incorporate big data analytics into the study of morality to gain a new way to gather information in natural settings about the structure of moral visions, large-scale moral behavioral patterns, and the relation between the two.

To our knowledge, only one study used the Moral Foundations Dictionary (MFD) to analyze real conversational data. The authors of that study used short-post social media to compare the accuracy of text analysis methods for detecting moral rhetoric and longer form political speeches to explore detecting shifts in that rhetoric over time [29]. They demonstrated how capturing moral rhetoric in text over time opens up new avenues for research such as assessing when and how arguments become moralized and how moral rhetoric impacts subsequent behavior. We used the MFD for this study, to provide a framework to “test” if we can use an existing data dictionary to analyze and explore moral values expressed in conversations by grouping words according to their morality framework.

## Methods

### Data

For this study, we used data from the Palliative Care Communication Research Initiative (PCCRI), a multi-site cohort study of naturally occurring inpatient palliative care consultations [30, 31]. The PCCRI was designed to understand the relation between clinical communication and patient-centered outcomes. The 6-month cohort data includes directly observed and audio-recorded palliative care consultations; patient/proxy and clinician self-report questionnaires both before and the day after consultation; post-consultation in-depth interviews; and medical/administrative records. The audio data for the PC consultations and follow-up interviews were converted to a transcription of text data for analysis.

The study data were collected for 231 hospitalized patients with advanced cancer who consulted with PC in two large academic medical centers in the United States. For our study we used the patient/proxy questionnaire for patients’ demographic information (age, gender, race, education, financial insecurity) and self-reported preference for comfort-directed care near EOL, and attitudinal variables such as distressing uncertainty, spiritual distress, emotional distress, religious affiliation (if any), and whether patients felt their spiritual needs were being met by their religious community or the medical system. We used verbatim transcriptions of the palliative care conversations to identify moral words using the MFD data dictionary described in the previous paragraph.

The psychologists who developed the MFD did this by classifying words in one of the five moral foundations, by vice or virtue. This results in 10 potential “dimensions” of moral words in the text: each of the 5 foundations with “vice” and “virtue” categories for each foundation.

### Text mining

We used 231 audio-recorded and transcribed inpatient PC consultations and data from baseline and follow-up patient questionnaires at two large academic medical centers in the United States. With these data, we identified different moral expressions using text mining techniques and natural language processing. The words that each patient or proxy said were combined into a single corpus of text. We included only text used by patients, not physicians or other members of the conversation. The corpus was then split into a list of individual words, which were set to lowercase and stemmed. Stop words, such as” and”,” the”, and” of”, were removed from each corpus to reduce the noise of the data.

First, we added up all the morality words used by the patient in a PC consultation, and counted, after pre-processing, the total number of words used by the patient as a proxy for the length of the conversation. We then disaggregated the words from the data dictionary to create the 10 different categories of moral terminology in the PC consultations. We created a matrix for all categories where a word from the Moral Foundations Theory Dictionary (MFD) occurred in a patient’s text, that patient was assigned a value of” 1″ for that word’s associated MFD category. The text mining process was performed with Python 3.7.3.

### Statistical analysis

After merging the data from the text with the data from the PC survey, we analyzed the data in a few steps, adopting an exploratory approach to test relations between underlying factors and moral expressions in the PC consultations.

First, we used latent class analysis (LCA) to classify the patterns of MFD expressions into mutually exclusive classes. LCA is based on the idea that a discrete latent variable accounts for observed associations between a set of indicators, such that, conditional on the latent class variable, these associations become insignificant [32]. A statistical indicator is simply an observed value of a variable, so that they can be used in statistical models to allow for meaningful comparisons and to show positive or negative change. “High quality” indicators are predicted by the latent variable, in this case “moral charge”, to have a probability near zero or one. Such indicators are generally necessary for model estimation and the interpretation of the latent classes.

The ten indicators in our analysis were created after the text mining phase: each one indicated whether a patient used a vice- or virtue- related word in one of the five dimensions of the MFT. In addition to the indicators (which are used for the actual classification) covariates were included in the model to explain class membership: age, gender, race, education, financial security and religion. We also included self-reported variables regarding patient’s spiritual needs, whether they reported emotional, spiritual or uncertainty-related distress, and preferences for comfort-directed treatment at EOL and looked at patterns of several of the attitudinal variables. Our analyses focused on preferences for comfort-directed EOL treatment; emotional, spiritual or uncertainty-related distress; and whether patients felt their spiritual needs were being met by (1) their religious community or (2) the medical system. EOL preference was defined by the answer to a survey question: “During the last few months of my life, I would prefer a plan of treatment that focused on my comfort and quality of life, even if that meant not living quite as long”, which is answered by a 5-point Likert scale.

The questions related to emotional feelings, also answered by 5-point Likert scales, included:

- Over the past 2 days, how much have you been bothered by emotional problems such as feeling anxious, depressed, irritable, or downhearted and blue.
- Over the past 2 days, how much have you been bothered by uncertainty about what to expect from the course of your illness?
- Over the past 2 days, how much have you felt at peace?

Questions related to spiritual needs included: “How much are your spiritual needs being supported by a religious community (like clergy or members of a congregation)?”, and: “How much are your spiritual needs being supported by the medical system (doctors, nurses and chaplains)?” where both were answered by “completely-quite a bit-moderately-slightly-not at all”.

Second, to explore which factors were associated with patients’ use of vice- or virtue-related words, and their use of words belonging to the 5 different foundations of morality, we used Poisson regressions. Age was a continuous variable, race was represented by a binary variable for “white”, education was categorical, and “financial security” was represented by a categorical variable: “When you think about the amount of income that you have available in a typical month, how often is it enough for things you really need like food, clothing, medicine, repairs to the home, and transportation?” – answered by “all the time”, “most of the time”, “some of the time”. We included a binary variable for “Christian” religion and one for “other religion” which included Judaism, Islam, Hinduism, Buddhism, and “other” from the survey data. We also controlled for the total amount of words used by the patient in the consultations, as a proxy for the length of the conversation.

## Results

### Descriptive statistics

The summary statistics are reported in Table 1. Three quarters of patients were above the age of 55 and half of the patients were female; 79% were White while the remaining 21% were either Black or Latino. About one third of the PC patients had a college degree or higher while half of the sample finished high school or had some years in college. One third of patients felt financially insecure, described by not having enough income in a typical month to pay for clothing, food or transportation. 67% of patients maintained a connection with Christianity, while 24% did not have a religion. Fewer patients connected with Judaism (2.1%), Islam (0.8%), Hinduism (1.3%) and Buddhism (0.4%); in total 9% of patients have some “other” religion than Christianity. About 69% strongly agreed or agrees that EOL treatment plan should focus on comfort and quality of life even at the expense of longevity, 22% were unsure and about 10% either disagreed or strongly disagreed. A little more than a third feels that their spiritual needs are supported by their religious community while one third beliefs that those needs are being met by the medical system. Almost half of patients have felt bothered by emotional problems such as feeling anxious, depressed, irritable, or downhearted and blue in the past 2 days. Also, about half of patients feels uncertain about the course of their illness and does not feel “at peace”.

**Table 1:** Summary Statistics

	Total sample (*n* = 231) % (*n*=)		Total sample (*n* = 231) % (*n*=)
**Age in years**		**Spiritual needs by rel. Community**	
< 55	27 (62)	Completely	23 (53)
55–70	45 (104)	Quite a bit	16 (37)
> 70	28 (65)	Moderately	8 (18)
**Gender**		Slightly	11 (25)
Female	49 (113)	Not at all	42 (97)
Male	51 (118)	**Spiritual needs by medical system**	
**Race/Ethnicity**		Completely	14 (32)
White	79 (182)	Quite a bit	15 (35)
Black or Hispanic	21 (49)	Moderately	14 (32
**Highest education**		Slightly	15 (35)
Did not graduate high school	16 (37)	Not at all	42 (97)
High school graduate or GED	29 (67)	**Emotional problems**	
Associate Degree / Technical School	27 (62)	Not at all	14 (32)
Bachelor’s Degree	12 (28)	Slightly	22 (51)
Masters or Doctorate Degree	16 (37)	Moderately	18 (42)
**Financial security**		Quite a bit	30 (69)
Secure	34 (79)	Extremely	16 (37)
Partially secure	28 (65)	**Bothered/prognostic uncertainty**	
Insecure	38 (88)	Not at all	10 (23)
**Religion**		Slightly	13 (30)
Christianity	67 (155)	Moderately	27 (62)
Other	9 (21)	Quite a bit	30 (69)
None	24 (55)	Extremely	20 (46)
**EOL preference for comfort directed treatment**		**Feeling “at peace”**	
Strongly Disagree	7 (16)	Completely	7 (16)
Disagree	3 97)	Quite a bit	15 (35)
Uncertain	22 (51)	Moderately	31 (72)
Agree	15 (35)	Slightly	27 (62)
Strongly Agree	54 (125)	Not at all	20 (46)

We continued our descriptive analysis looking at the use of MFD words. We found that about half of the patients did not use any of the MFD words at all. For those who did use MFD words, we looked at the number of words they used per category of the Moral Foundations Theory. Table 2 provides an overview of the MFD words used in each of the 10 dimensions of morality: five moral foundations times two sub-categories (vice and virtue) per foundation. It also reports the total number of morality words used by patients, the total number of words used in the consultation and their relative frequency.

**Table 2:** Number of moral words used in the PC consultations, per dimension of the MFD and “virtue” and “vice”

	Harm virtue	Harm vice	Fairness virtue	Fairness vice	Ingroup virtue	Ingroup vice	Authority virtue	Authority vice	Purity virtue	Purity Vice
**Total virtue across foundation**	112		52		121		115		34	
**Total vice across foundation**		97		4		5		2		38
**Total**	**209**		**56**		**126**		**117**		**72**	

### Latent class models

After identifying how many moral words were being used in the different dimensions of morality and their vice-virtue subcategories, we explored the results of the latent class analysis (LCA). The dependent variable was “moral charge” defined by the number of moral words used in the PC consultation; the indicators were the 10 morality categories. First, we determined the number of latent classes. Table 3 reports the fit of the models using a different number of classes.

**Table 3:** LCA results: Model fit, class size and profiles in 2-class solution

	LL	BIC (LL)	L^**2**^	df	No. of parameters	
**1-Class**	− 1210.1	2474.7	854.5	221	10	
**2-Class**	− 1079.7	2273.8	593.6	210	21	
**3-Class**	− 1073.0	2320.1	580.1	199	32	
**4-Class**	− 1068.1	2370.1	570.3	188	43	
**5-Class**	− 1064.4	2422.7	563.0	177	54	
					**Class 1**	**Class 2**
**Size (%)**					**68.3**	**31.7**
**Indicators (# words)**						
Harm-Virtue (Wald = 67, *p* < 0.00)					0.25	0.99
Harm-Vice (Wald = 33, *p <* 0.00)					0.20	0.90
Fairness-Virtue (Wald = 42, *p <* 0.00)					0.06	0.57
Fairness-Vice (Wald = 0.3, *p <* 0.62)					0.01	0.03
Ingroup-Virtue (Wald = 56, *p* < 0.00)					0.23	1.17
Ingroup-Vice (Wald = 19, *p <* 0.00)					0.00	0.06
Authority-Virtue (Wald = 71, *p <* 0.00)					0.14	1.27
Authority-Vice (Wald = 4, *p* = 0.04)					0.00	0.02
Purity-Virtue (Wald = 24, *p <* 0.00)					0.01	0.43
Purity-Vice (Wald = 8, *p* = 0.01)					0.01	0.10
**Covariates**						
**Gender (%)**						
Male					52	48
Female					48	52
**Age (%)** (Wald = 15, *p <* 0.00)						
< 22					20	17
23–30					21	18
31–39					22	21
40–45					20	18
46–61					17	26
**Race and Ethnicity (%)** (Wald = 8, *p <* 0.00)						
Black or Hispanic					20	25
White					80	75
**Education (%)** (Wald = 4, *p =* 0.04)						
Did not graduate high school					18	11
High school graduate or GED					33	19
Associate Degree / Technical School					24	31
Bachelor’s Degree					12	15
Masters or Doctorate Degree					13	24
**Financial Security (%)** (Wald = 1, *p =* 0.23)						
None of the time					8	8
Some of the time					25	28
Most of the time					29	27
All of the time					38	37
**Spiritual Needs supported (%)** (Wald = 3, *p =* 0.08)						
Completely					22	19
Quite a bit					18	23
Moderately					17	21
Slightly					9	8
Not at all					28	26
Missing					6	2
**Emotional Distress (%)** (Wald = 0.4, *p* = 0.51)						
Completely					17	17
Quite a bit					22	19
Moderately					12	20
Slightly					21	26
Not at all					22	17
missing					6	1
**EOL preference focus comfort-directed treatment (%)** (Wald = 0.4, *p* = 0.55)						
Strongly disagree					8	4
Disagree					3	2
Not sure					23	18
Agree					12	19
Strongly agree					50	56
missing					4	1
**Total words used (excluding stop words) (%)** (Wald = 22, *p <* 0.00)						
< 32					29	0
33–73					29	0
74–110					28	3
111–151					13	36
152–197					1	61
**Christian (%)** (Wald = 8, *p =* 0.01)						
0					32	28
1					68	72
**Other religion (%)** (Wald = 7, *p =* 0.01)						
0					93	86
1					6	14

*LL* log-likelihood, *L*^*2*^ likelihood-ratio chi-squared statistic, *df* degrees of freedom

*Results for indicators represent percentages; numbers for covariates represent counts (n)*

We considered theoretical interpretability and compared the statistical tests of model fit using models for one to five possible latent classes. Table 3 illustrates that the likelihood decreased slightly when moving from two classes to three classes while the Bayesian Information Criterion (BIC) of the 2-class model was lowest, suggesting the 2-class model provides the optimal balance between model fit and model complexity.

Table 3 also illustrates the profiles of the two latent classes, including the class sizes, the indicators and the covariates mentioned in the previous section. The Wald test statistics indicate that 9 of the 10 indicators are highly significant and thus classify the two groups, except the indicator “Fairness-Vice” (Wald 0.2484, *p* = 0.62). Overall, the two classes can be interpreted as one in which individuals use many morality words (31.7% of the sample of patients) and one where moral terms occur infrequently (68.3%). Individuals in the first class use some words in the Harm-virtue, Harm-vice and Ingroup-virtue dimensions, but not many in the other dimensions of morality.

Except for gender, financial security and preferences for comfort-directed treatment near EOL, all exogenous variables (age, white race, education level, Christian, other religion, emotional, spiritual, and uncertainty-related distress and spiritual needs) are associated with class membership. Being female, feeling financially secure and preferring comfort-directed treatment near EOL are independent of class membership. There are slightly more males (52% vs 48%) in the class using fewer moral words, and more younger patients and more Whites (80% vs 75%). Overall, among patients in class 1, there were fewer Christians (68% vs 72%) and fewer patients with another religion (6% vs 14%).

### Poisson models

Following the LCA, we looked at which variables were related to the number of morality words used. First, we explored the variables associated with vice and with virtue, to see if any of the individual characteristics, religious affiliation or attitudes were related to the use by patients of virtue- versus vice-words. The data followed a Poisson distribution: the use of virtue and vice words could be treated as rare events, since many patients did not use MFD words at all. As the Poisson distribution assumes that the mean and variance are the same, we tested the fit of a Poisson model versus Negative Binomial models. The likelihood ratio test is a test of the over dispersion parameter alpha: when alpha is zero, the more flexible negative binomial distribution is equivalent to a Poisson distribution. In our case, alpha was not significantly different from zero, suggesting the Poisson distribution was appropriate, both for virtue and for vice, so we used Poisson regressions to estimate the amount of moral rhetoric in PC consultations. We also used a Vuong test of the zero-inflated model versus the standard Poisson model and found that the excess zeros should not be modeled independently. We used robust standard errors for the Poisson models [33]. In all Poisson models, we controlled for the length of the conversation by normalizing based on the total number of words used by the patient in the consultation.

Table 4 reports the results of the virtue and vice models. We found that being White and Christian was, on average, associated with using fewer words in the virtue categories (− 0.46 (*p* = 0.09); − 0.42 (*p* = 0.05)). Patients who had been increasingly bothered by emotional problems such as feeling anxious, depressed, irritable, or downhearted and blue, felt more uncertain about their prognosis or felt less “at peace”, used more moral terms in the “vice” category of the MFD (*p* < 0.01).

**Table 4:** Poisson models: Words Used in Virtue and Vice, marginal effects

	Virtue	Vice
Age	0.0072 (0.0067)	−0.0043 (0.0034)
Female	− 0.0781 (0.1991)	−0.0389 (0.1079)
White	−0.4625* (0.2693)	− 0.1159 (0.1267)
Education	0.0470 (0.0637)	0.0245 (0.0355)
Financial security	0.0628 (0.1017)	−0.0485 (0.0508)
Christian	−0.4236** (0.2157)	0.0024 (0.1131)
Other religion	−0.3908 (0.3453)	− 0.2195 (0.2540)
EOL preference for comfort-directed treatment	0.0297 (0.0887)	−0.0155 (0.0520)
Spiritual needs supported	−0.0443 (0.0507)	0.0226 (0.0239)
Psychospiritual Distress	0.0251 (0.0298)	0.0468*** (0.0180)
Total words used	0.0037*** (0.0003)	0.0011*** (0.0001)
Observations	231	231

*Results report marginal effects – representing the change in the number of words used. Robust standard errors in parentheses*

*** p < 0.01, ** p < 0.05, * p < 0.1

After establishing that emotional distress, white race and Christianity were associated with the use of virtue and vice words by patients, we were interested in which variables were related to using words in each of the five distinct morality foundations described by the MFT (merging the vice and virtue sub-categories per dimension).

Table 5 reports the results of Poisson models estimating the use of words in these dimensions. We found that being white was also associated with the use of fewer words in the “Care/Harm” foundation (− 0.25, *p* = 0.07); and being Christian was related to using fewer “Loyalty/Betrayal” words (− 0.29, *p* < 0.01). Feeling more emotional, spiritual or uncertainty-related distress was associated with more words in “Care/Harm” and “Sanctity/Degradation”.

**Table 5:** Poisson models: Words Used in 5 Foundations, marginal effects

	(1) Harm	(2) Fair	(3) Loyal	(4) Authority	(5) Sanctity
Age	−0.0022 (0.0043)	−0.0008 (0.0020)	0.0019 (0.0032)	0.0019 (0.0032)	0.0008 (0.0026)
Female	−0.0622 (0.1191)	0.0158 (0.0677)	0.0295 (0.0894)	−0.0078 (0.1110)	−0.0712 (0.0646)
White	−0.2445* (0.1361)	−0.0228 (0.0792)	− 0.1513 (0.0972)	−0.0839 (0.1442)	− 0.0801 (0.0886)
Education	0.0063 (0.0423)	0.0425* (0.0243)	−0.0587** (0.0306)	0.0789** (0.0335)	0.0064 (0.0221)
Financial security	−0.0774 (0.0721)	−0.0006 (0.0347)	0.0216 (0.0427)	0.0866 (0.0656)	−0.0015 (0.0369)
Christian	0.0821 (0.1392)	−0.0829 (0.0646)	−0.2852*** (0.1061)	− 0.0914 (0.1128)	−0.0348 (0.0721)
Other religion	−0.2938 (0.2730)	−0.0691 (0.1111)	− 0.1738 (0.1274)	0.1159 (0.1337)	− 0.2405 (0.1551)
EOL pref. Focus comfort care	− 0.0331 (0.0524)	−0.0198 (0.0267)	0.0528 (0.0428)	−0.0230 (0.0416)	0.0517 (0.0439)
Spiritual needs supported	0.0017 (0.0257)	0.0272* (0.0152)	−0.0312* (0.0194)	−0.0254 (0.0273)	0.0056 (0.0133)
Feeling emotional	0.0415** (0.0217)	−0.0152 (0.0100)	0.0031 (0.0149)	0.0209 (0.0171)	0.0229** (0.0116)
Total words used	0.0016*** (0.0001)	0.0005*** (0.0001)	0.0012*** (0.0001)	0.0010*** (0.0001)	0.0005*** (0.0001)
Observations	231	231	231	231	231

*Results report marginal effects – representing the change in the number of words used. Robust standard errors in parentheses*

*** p < 0.01, ** p < 0.05, * p < 0.1

In addition, we found that patients who were higher educated used, on average, slightly more words in the “Fairness/Cheating” foundation (0.04, *p* = 0.08) and “Authority/Subversion” (0.08, *p* = 0.02), and fewer words in “Loyalty/Betrayal” (− 0.05, *p* = 0.05). Interestingly, the more patients felt that their spiritual needs were being supported by their religious community or the medical system, the more words they would use in the “Fairness/Cheating” foundation (0.03, *p* = 0.07), but fewer words in “Loyalty/Betrayal” (− 0.03, *p* = 0.10).

## Discussion

This study used data from transcribed palliative care consultations to identify moral expressions used by hospitalized patients with advanced cancer and to analyze if individual characteristics, religion, self-reported EOL preferences, spiritual needs and emotional distress were associated with (differences in) the moral lexicon as determined by the moral foundation dictionary (MFD) corpus. We found in our LCA that about two thirds of patients use few or no morality words at all while about a third does use a lot of moral rhetoric. Employing the MFD, which distinguishes five moral foundations and vice and virtue subcategories within each dimension, we found that being White and Christian were both associated with the use of fewer words in the “virtue” category and more emotional distress were associated with the use of more “vice” words. These factors were also related to the use of words in Care/Harm, Loyalty/Betrayal, and Sanctity/Degradation dimensions of the MFT. We also found that education level was related to the use of words in Fairness/Cheating, Loyalty/Betrayal and Authority/Subversion. To what extent patients stated that their spiritual needs were being supported by religious community or medical system was also associated with moral rhetoric used in the Fairness and Loyalty foundations.

There are a number of limitations in this study. First, our analysis assumes that the MFD is a correct tool to identify morality, and in particular the five different foundations identified in the MFT. However, the data dictionary is relatively novel and has not been tested very often empirically, other than the study mentioned further above. Second, our study results may not be generalizable to other populations of patients. There are further limitations associated with the bag of words-approach that we used in the text mining phase of the study. A disadvantage is that it limits the context of the conversation and loses the order of specific information. Bag-of-words requires supervised machine learning which entails modeling linguistic knowledge through the use of dictionaries containing words that are tagged with their semantic orientation [33]. We used the existing MFD data dictionary which was created by others and we accept the classification of the English words to identify morals as a given.

The most important piece of our study was to adopt a plurality perspective to morality, and therefore we wanted to distinguish between different types of morality words used by different patients. In order to further explore the differentiation of moral terminology and evaluate which factors are related to specific moral terms, we would need more data as for some groups we did not have enough words in some MFT foundations. Based on our analysis, for example, we found that having any religion mattered but we could not differentiate enough between morality among several religions because of sample size issues.

## Conclusions

This study is among the first to use text data from a real-world situation to extract information regarding individual foundations of morality. It is the first to test empirically if individual moral expressions are associated with individual characteristics, attitudes and emotions. The results of this study are relevant to those who seek to improve the quality of communication in order to achieve better and more values-concordant treatment at EOL.

Some of our findings may be relevant for a broader context. We found that those who feel that their spiritual needs are being met tend to use more moral language than those who do not. This study gives rise to the further development of conversation science which can be used by physicians to align moral and other sensitive aspects of PC consultations [34]. For example, it may be helpful to differentiate prognosis communication with respect to patients a-priori moral or spiritual values which may influence their EOL preferences. More research would be needed to establish the exact relationship between (any) religious affiliation and spirituality on the moral dimensions of conversations, in palliative care and in a broader societal context.

## Data Availability

The datasets generated and analyzed during the current study are not publicly available due to the consenting process but are available from the corresponding author on reasonable request.

## Abbreviations

EOL: End of Life
LCA: Latent Class Analysis
MFT: Moral Foundation Theory
MFD: Moral Foundations Dictionary
NLP: Natural Language Processing
PC: Palliative Care
PCCRI: Palliative Care Communication Research Initiative
